# P-1672. Emergency preparedness and integrated surveillance potential of a rapid COVID-19 and TB diagnostic in Bangladesh: An adaptive response in health system governance

**DOI:** 10.1093/ofid/ofaf695.1846

**Published:** 2026-01-11

**Authors:** Muhammad Asaduzzaman, Farzana Zaman

**Affiliations:** University of Oslo, Oslo, Oslo, Norway; National TB control Program, Directorate General of Health Services, Bangladesh, Dhaka, Dhaka, Bangladesh

## Abstract

**Background:**

The impact of the COVID-19 situation, coupled with the continual emergence of new variants, had significantly disrupted not only the local health system but also the aviation system and global public health. Subsequently, many unprecedented challenges have been observed at the health system management of resource poor settings, notably inadequate airport quarantine facilities and lack of highly sensitive yet rapid and cost-effective testing. Therefore, we aimed to present a cost-effective and timely alternative diagnostic and quarantine facility for the incoming travelers in such settings.

**Figure 2.**
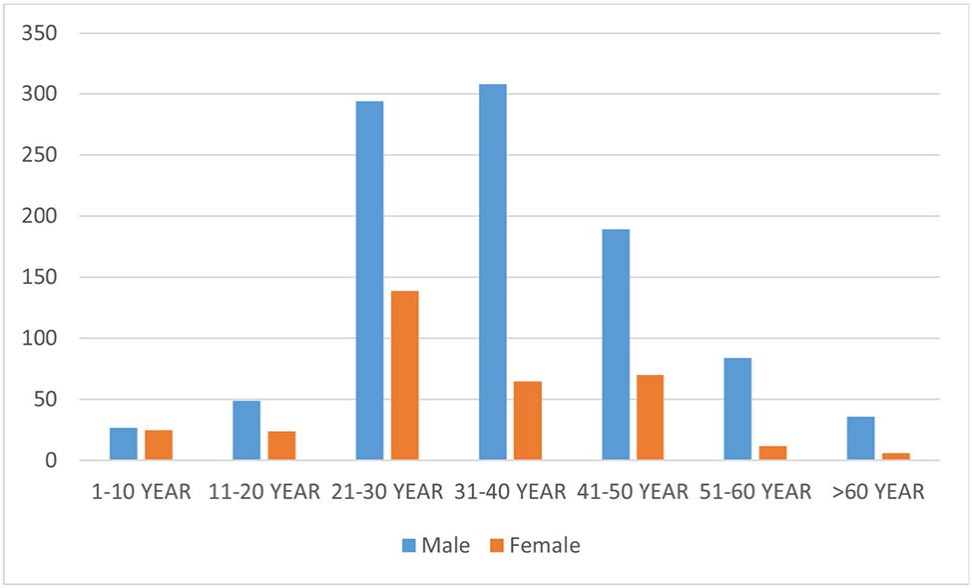
SARS-COV-2 positive patients from different countries

**Methods:**

We conducted a cross-sectional serosurvey from December 15, 2020 to November 30, 2021 for inbound travelers to Bangladesh from different countries. These impoverished travelers (unable to afford hotel quarantine) were quarantined in Hajj camp (a government facility near the airport reserved for muslim pilgrims) with full coverage of food and lodging at subsidized cost and free testing by GeneXpert, which is capable of Nucleic Acid Amplification Test (NAAT) for both COVID-19 and TB.

**Figure:**
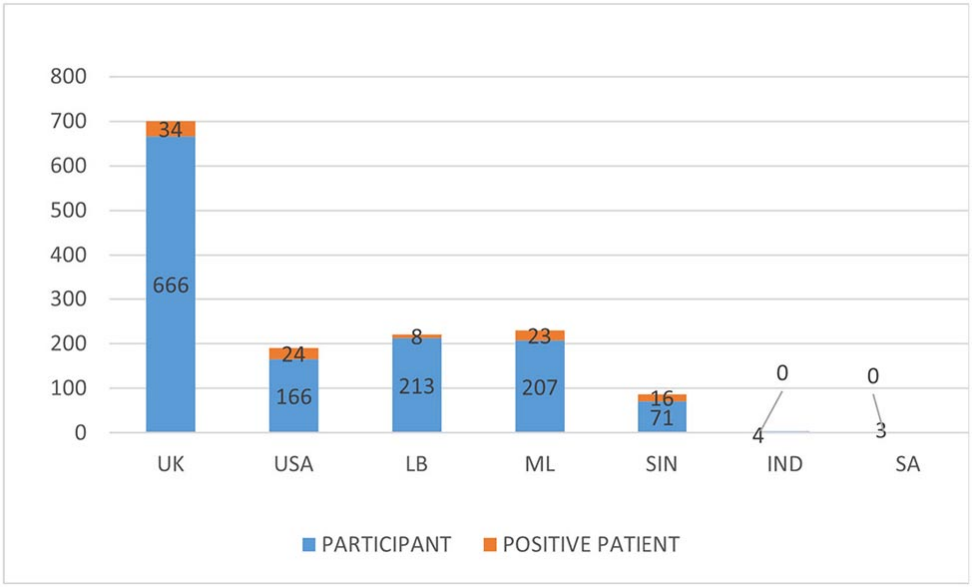

**Results:**

The majority of our study participants were male (74.32%), mainly in the 20-40 year age group. Among 1328 participants, 106 (7.98%) tested positive for SARS-COV-2, with 72 being male. The highest infection rate was observed in travelers from Singapore (22.53%, n=16/71), followed by USA and Malaysia. The NAAT result processing time was only 1 hour, enabling 92% (1222 participants) to travel to their destinations on the same day. The positive cases were kept in quarantine for further assessment.

**Conclusion:**

Our study findings endorse the equitable access to COVID-19 diagnostics for disadvantaged groups and optimizing the effective utilization of limited health system resources. Due to high sensitivity and specificity, NAAT is also recommended by WHO for diagnosis of acute COVID-19 cases. Therefore, our research also emphasizes on the potential to equip hard to reach laboratory facilities by GeneXpert for both TB and COVID-19 diagnostics to ensure fair and sustainable health systems, particularly in regions facing the dual burden.

**Disclosures:**

All Authors: No reported disclosures

